# Postural control in total knee arthroplasty patients with patellofemoral pain syndrome before and six months after re-operation

**DOI:** 10.1186/1757-1146-5-S1-O33

**Published:** 2012-04-10

**Authors:** Helena Gapeyeva, Tiit Haviko, Aare Märtson, Herje Aibast, Jaan Ereline, Mati Pääsuke

**Affiliations:** 1 Institute of Exercise Biology and Physiotherapy, University of Tartu, Tartu 51014, Estonia; 2 Department of Traumatology and Orthopaedics, University of Tartu, Tartu 51014, Estonia

## Background

Although excellent long-term clinical results have been reported for the total knee arthroplasty (TKA), 37% of patients have limited functional improvement one year after the surgery [1]. Patients with a clinical presentation of anterior knee pain could be diagnosed with patellofemoral pain syndrome (PFPS). Modified clinical classification of PFPS patients includes two main groups: with malalignment and with muscular dysfunction [2]. The aim of the study was to compare postural stability characteristics in TKA patients with PFPS before and six months after re-operation.

## Materials and methods

Twelve patients aged 59-77 years with PFPS following unilateral TKA participated in the study. Pre-TKA, all patients had primary degenerative knee OA in stage III or IV (Kellgren-Lawrence Scale) and were scheduled for the first TKA. Duration of pain before TKA was 9.3±2.5 years and re-operation due to PFPS was performed 18.8±3.5 months later. Patella malalignment was noted in eight patients and patella altered position in three patients. Static standing balance was assessed by centre of foot pressure (COP) sway registered during 30 s quiet bipedal standing with eyes open on twin force plates *Kistler* 9286A (Switzerland) using S*way* software of motion analysis system *Elite* (BTS S.p.A., Italy). Plantar pressure distribution was recorded by *Digital Biometry Scanning System* and *Milletrix* software (DIASU, Italy). Data are means and standard errors of means (±SE).

## Results

COP sway trace radius of PFPS leg was significantly shorter 6 month after re-operation as compared before it (5.91± 0.48 and 4.22 ± 0.22 mm, respectively, p=0.007). No significant difference was found in COP trace length and velocity as compared pre- and post-surgery data (p>0.05). Significant decrease of plantar pressure distribution in forefoot of PFPS leg was noted (p<0.05, Table 1).

**Table 1:** Plantar pressure distribution (weight ratio %) in TKA patients with PFPS before and 6 months after re-operation

Characteristics		Before re-operation	After re-operation	p
FOREFOOT	PFPS leg	66.37 ± 4.90	51.55 ± 1.64	0.021

	Non-PFPS leg	64.15 ± 6.47	52.16 ± 3.40	NS

REARFOOT	PFPS leg	44.85 ± 1.75	51.54 ± 1.86	0.021

	Non-PFPS leg	49.16 ± 3.36	47.84 ± 3.40	NS

## Conclusions

Main findings of our study were: (1) postural control in TKA patients with PFPS significantly improves (and 2) re-distribution of plantar pressure from forefoot to rearfoot in PFPS leg takes place 6 months after re-operation. The link between the segmental configuration of the lower limbs was described [3] and the importance of paying attention to balancing of the PF soft tissues was emphasized in studies of PF pain after TKA [4].
